# A common methodological phylogenomics framework for intra-patient heteroplasmies to infer SARS-CoV-2 sublineages and tumor clones

**DOI:** 10.1186/s12864-021-07660-9

**Published:** 2021-11-16

**Authors:** Filippo Utro, Chaya Levovitz, Kahn Rhrissorrakrai, Laxmi Parida

**Affiliations:** IBM Research, T.J. Watson Research Center, Yorktown Heights, USA

**Keywords:** Tumor evolution, Clonal evolution, Phylogeny, COVID-19

## Abstract

**Background:**

All diseases containing genetic material undergo genetic evolution and give rise to heterogeneity including cancer and infection. Although these illnesses are biologically very different, the ability for phylogenetic retrodiction based on the genomic reads is common between them and thus tree-based principles and assumptions are shared. Just as the different frequencies of tumor genomic variants presupposes the existence of multiple tumor clones and provides a handle to computationally infer them, we postulate that the different variant frequencies in viral reads offers the means to infer multiple co-infecting sublineages.

**Results:**

We present a common methodological framework to infer the phylogenomics from genomic data, be it reads of SARS-CoV-2 of multiple COVID-19 patients or bulk DNAseq of the tumor of a cancer patient. We describe the Concerti computational framework for inferring phylogenies in each of the two scenarios.To demonstrate the accuracy of the method, we reproduce some known results in both scenarios. We also make some additional discoveries.

**Conclusions:**

Concerti successfully extracts and integrates information from multi-point samples, enabling the discovery of clinically plausible phylogenetic trees that capture the heterogeneity known to exist both spatially and temporally. These models can have direct therapeutic implications by highlighting “birth” of clones that may harbor resistance mechanisms to treatment, “death” of subclones with drug targets, and acquisition of functionally pertinent mutations in clones that may have seemed clinically irrelevant. Specifically in this paper we uncover new potential parallel mutations in the evolution of the SARS-CoV-2 virus. In the context of cancer, we identify new clones harboring resistant mutations to therapy.

**Supplementary Information:**

The online version contains supplementary material available at (10.1186/s12864-021-07660-9).

## Background

Deep sequencing genomic datasets contain intricate details that can be mined to reveal intra-patient heterogeneity present in disease states. The classic example that has been explored is the heterogeneity present in cancer, whether it be within a single tumor, across a patient’s metastatic sites, or a tumor’s evolution in response to treatment over the course of a disease. Interestingly, these same principles of heterogeneity can be explored in other scenarios that have similar sequencing data demonstrating different variant frequencies, including SARS-CoV-2 virus causing the COVID-19 infection. Evidence in several studies have highlighted the intra-host genomic diversity of SARS-CoV-2 [1–5]. As in cancer, the presence of different, heterogenic reads in a COVID-19 patient assumes the existence of multiple sublineages, or subclones, rather than the occurrence of recombination. The genetic evolution giving rise to heterogeneity is a common characteristic of all aspects of disease that contain genetic material, including cancer and infection. This common thread of increased heterogeneity involves many of the same processes. Once these assumptions are established, the same tools and methodologies that are used to analyze tumor heterogeneity can be applied with a level of confidence to SARS-CoV-2 datasets.

**Implications of viral heteroplasmy in COVID-19 patients.** The novel SARS-CoV-2 coronavirus that appeared in the city of Wuhan, China, in late 2019 has caused a large scale COVID-19 pandemic, spreading to more than 70 countries. Broad sequencing efforts have been made in an effort to understand the natural evolution of this virus. Several studies published with SARS-CoV-2 sequencing data reveal different viral allele frequencies in the same patient. The most likely explanation for the presence of intra-patient heterogenic viral reads is the existence of different viral strains rather than recombination since the probability of a fully functional single stranded virus emerging after entering a cell and its subsequent disassembly and reassembly into a virion with a different sequence is low [6]. Multiple viral strains infecting the same host has enormous clinical implications in terms of treatment, epidemiology, and the potential to overcome the pandemic and thus needs to be considered and analyzed. Variations in viral strains can harbor different resistance mechanisms, levels of transmissibility, response to therapy, and explain the large variation of symptomology. Even more important, treatment and vaccine success would rely on targeting the collection of strains present and not simply targeting one. It is for these reasons it is imperative that the research community consider the likely scenario that patients are coinfected with multiple strains.

**Implications of heterogeneity in tumors of cancer patients.** The presence of multiple tumor clones in the same patient has significant treatment implications. Multiple mechanisms of resistance can exist in separate clones [7]. Drug targets can ‘disappear’ or develop over time [8, 9]. Alternate pathways can be inhibited by the introduction of new alterations [10]. Even gross phenotypes can change due to underlying genomic changes [11]. Thus, it is imperative that we continue to monitor patient tumor evolution over the course of a disease in order to optimize treatment protocols. Parallels of tumor evolution have been drawn to that of human evolution and thus similar tools and algorithms are being applied and adjusted to analyze cancer [12]. Phylogenetic trees are being constructed to capture the change occurring during the disease while subclonal structure is identified and analyzed for clinically relevant changes. Several algorithms have been proposed to capture tumor evolution using single cells [13, 14] however these tools do not account for tumor heterogeneity and thus do not pick up on all clones present in a given tumor. Most algorithms consider bulk tumor samples which has the advantage of integrating genetic information from many tumor cells but are challenged by the need to deconvolve the mixture of clones present in any given biopsy [15–21]. Studies have also shown that determining which tree amongst the multitude that are possible is a non-trivial problem [22]. Several of these methods have been adapted for multi-site sample integration but are not specific for longitudinal data [16, 18, 19, 23]. More recently there have been several tools developed that do integrate longitudinal (multi-time) sampling [24, 25]. Although these models are more accurate, they still are limited by their inability to deal with samples with large mutational burdens and are not designed for multi-site samples.

**Concerti overview** An informative analysis for SARS-CoV-2 would require a method to be able to perform fine-grain evaluations with the ability to differentiate between viral sequences. In addition, the method would need to be able to analyze longitudinal data to capture when co-occurrence transpires. In cancer, sequential liquid biopsies over the course of disease are becoming more common given the ease of collection, lower cost, and greater ability to describe the complete disease profile vs. a subset of mutations present in distinct lesions. Therefore, it is imperative to establish tools that can manage/deconvolve mixed clonal samples, integrate multi-site and longitudinal sampling, and analyze large numbers of mutations with the same level of accuracy as low burden samples. We introduce Concerti^1^, a tool for inferring disease evolution phylogenies, at genomic scales, from multiple sites and multiple longitudinal DNA sequencing samples. One of the unique features of Concerti is that it generates *time-scaled trees*, i.e., trees aligned to actual time scales that capture not only the birth and death of clones, but also acquisition of alterations within the same clone. Concerti uses almost exclusively discrete optimization methods and has the flexibility to provide multiple possible solutions suggested by the patient data. To help with the interpretation of the results, the solutions are ordered by decreasing likelihood. Due to the absence of benchmark data, it is hard to perform a precise comparison of the different tools reported in literature. We provide in Table 1 a succinct summary of the capabilities of eight classes of exemplar tools and highlight the elements of uniqueness in each approach.

**Table 1:** Exemplar phylogenetic methods

Method	Input data	Data mode	CNV	Multi-time data	Multi-site data	Multi-patient data	Genomic scale	Time-scaled tree
SCITE [13]	single cell	single value	✗	✗	✗	✗	N/A	✗
Pyclone [20]	bulk data	single value	✗	✗	✓	✗	✗	✗
CITUP [18]	bulk data	single value	✗	✗	✓	✗	✗	✗
Calder [25]	bulk data	single value	✗	✓	✗	✗	✗	✓
VERSO [4]	bulk data	single value	✗	✗	✗	✓	✓	✗
BEAST [26]	bulk data	single value	✗	✗	✗	✓	✓	✗
ClonalTREE [27]	bulk data	single value	✗	✓	✓	✗	✓	✗
PhylogicNDT [24]	bulk data	dist.	✓	✓	✓	✗	✓	✗
Concerti	bulk data	dist.	✓	✓	✓	✓	✓	✓

We demonstrate the accuracy of Concerti by reproducing and expanding on known results from literature. In particular, we confirmed the results reported in [4] for the viral evolution model and expanded on them by discovering new homoplasies. While for the tumor evolution model using whole-exome sequencing data from patients [7, 28], Concerti constructs phylogenetic trees that accurately describe a tumor’s evolution while simultaneously highlighting new post-treatment subclones that likely confer resistance and may serve as new potential drug targets.

## Method

**Viral Evolution Model**. For computational purposes, we assume that all the virions of the same lineage have the same set of alterations (with respect to a reference). Since there is evidence of intra-patient variations with a wide range of allele frequencies [4, 6], we postulate that there is heteroplasmy due to possibly multiple sublineages evolving in this micro-environment. Since the coronavirus is a non-segmented positive RNA virus, we further postulate that it is very unlikely that any recombination occurs during the virus’s life cycle: attachment and entry, replicase protein expression, replication and transcription, assembly and release [29].

**Tumor Evolution Model**. We assume the following model: tumors arise from an altered cell, accumulating additional alterations over time. These changes give rise to populations of cells termed in literature as *clones*. For computational purposes, we assume that all the cells in a clone have the same set of alterations. Furthermore, these clones may alter further over time. Thus multiple clones co-exist in a tumor and some may have an evolutionary advantage over the others within the tumor environment, allowing for growth or shrinkage of a clone over time.

**Terminology**. The absence of recombination and the accumulation of variants over time are the two salient factors that facilitate a common methodology for inferring evolution in both models. Furthermore for the tumor evolution model, the inferencing may be based on single or multiple DNA sequencing samples: the latter can be *multi-time*, i.e., at multiple timepoints, or can be *multi-site*, i.e., from different lesions possibly collected at the same time. We use the term *data point* for multi-patient (COVID-19) and multi-time, multi-site (cancer). The term *alteration* is applicable to any genetic event including, but not limited to, mutation, single nucleotide variant, copy number variant, etc. In this manuscript CCF (Cancer Cell Fraction) denotes the fraction of cancer cells bearing an alteration in a cancer sample [30]. For the purposes of our algorithm, CCF and VAF (Variant Allele Frequency) are indistinguishable and the precise method of determining alteration frequencies is outside the scope of this paper. For clarity of exposition we use VAF to represent VAF or CCF and SNV to represent all alterations.

**Fig. 1:**
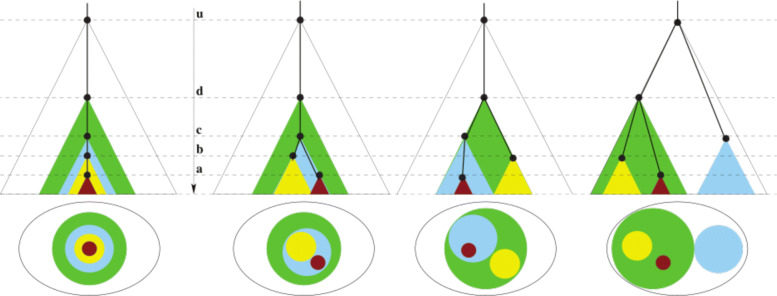
Let U (white), D (green), C (cyan), B (yellow), A (brown) be pseudoclones with prevalence values: 1.0≥*u*>*d*>*c*>*b*>*a*>0 respectively. The top row shows 4 possible evolution trees where the time axis is the *molecular clock*. The bottom row shows the single time-point “fishplot” as appropriately stacked disks. Notice that the leftmost phylogeny suggests that there exist some cells/virion with both A and B alterations while all the other three suggest that there exists no such cell/virion

Furthermore, in the context of cancer, it is important to note the distinction between *clones* and *pseudoclones*. Clone is a biological entity described as a population of indistinguishable cells. For our purposes, the nuclear DNA is identical for the population and thus a clone can be defined by a set of SNV’s. A pseudoclone on the other hand is a subset of these SNVs. In practice they are a maximal collection of SNVs with identical (or similar) VAF values [19, 20, 25]; this value is termed *prevalence* in this paper. This collection of SNVs is meaningful under the assumption that identical VAF values implies these SNVs co-occur in a cell. Note that the converse is not necessarily true, i.e., multiple SNVs within a cell may have varied VAF values. Thus pseudoclone is an algorithmic artefact while a clone is simply the union of some finite pseudoclones. For example in Fig. 1, the distinct colors denote the different pseudoclones, but the biological clone is the union of all the subclones in the path to the root of the evolutionary tree. Hence the (biological) brown clone in the leftmost tree is actually the union of the SNVs that define the brown pseudoclone, the yellow pseudoclone, the cyan pseudoclone and the green pseudoclone. Hence for mathematical preciseness, we use the term pseudoclones in the Method sections, yet to avoid clutter, we use the term clone in place of pseudoclone in the Results and discussion section.

With a slight abuse of terminologies, a pseudoclone and clone corresponds to a sublineage and lineage, respectively, in the context of virions. To avoid clutter, we use the terms sublineage and lineage interchangeably.

### Method assumptions

We make the following assumptions.

#### **Assumption 1**

**[Infinite Sites Model]** A majority of the alterations satisfy the following:

1. irreversible, i.e., once the alteration occurs the reverse of turning it back to its original state does not occur (no back mutation).
2. unique, i.e., the same alteration does not occur elsewhere in the tumor (no parallel mutation).

The topology of evolution is a tree. Although Occam’s Razor Principle suggests the perfect phylogeny assumptions used most commonly in literature [31], it is important to note that some exceptions to this property of alterations may occur in practice, especially when modeling biology. One such example is the presence of parallel mutations known as homoplasy. In order to capture this natural phenomenon in our trees, we handle this violation of perfect phylogeny as an exception in our algorithm.

#### **Assumption 2**

**[Alteration distribution]** Most of the alterations follow i.i.d. (uniform) distribution.

Again, for algorithmic purposes, it is reasonable to assume that tumor clones would follow the same principles as the individual alteration. But a clone, unlike an alteration, may die, i.e., may be selected against and overrun by other clones. So a clone may change in composition over time, i.e. more alterations can be added to the clone (but, not removed due to Assumption 1). Various selection pressures are in effect on the different clones, whose effect is manifested in the size of the clones: the clone can either grow or shrink in size reflected as an increasing or decreasing VAF value respectively. Thus the following:

#### **Assumption 3**

**[Tumor Clone dynamics]** Over time, a clone may

1. change in composition / size (additional alterations but not lose alterations)
2. change in prevalence values (increase or decrease)
3. die or a new clone may be born.

### Method overview

**Input** The input may come as one of two forms for both SNVs and CNVs. Single value VAF and CCF are taken as a matrix of data points (multi-patient, multi-time, or multi-site) by SNV. CCF distributions are received as a dataframe with data point, SNV, and CCF distribution discretized into *x* bins (*x*=100), which relates to the confidence associated to the CCF by the originating algorithm. All values are continuous [0,1]..

See Fig. 2 for an overview of Concerti. Based on our assumptions, the method has two major phases.

1. **Phase I**. We first identify the pseudoclones across all the data-points. However, the pseudoclones are not identical due to the clonal dynamics (Assumption 3).
  - Due to Assumption 3(3), we gate the alterations that are present in all the samples. This results in some alterations being filtered out and refer to this step as Negative Selection in the outline. We cluster these filtered alterations separately for each sample.

**Fig. 2:**
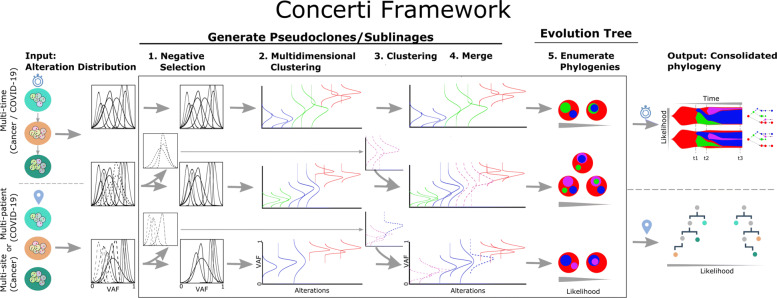
Schematic of the Concerti Framework. Given a set of multi-patient (COVID-19) or multi-site, multi-time (cancer) genomic samples, the algorithm analyzes the underlying alteration frequency distribution as input and performs a (1) negative selection to filter *appearing* alterations. A (2) multidimensional clustering is done to identify pseudoclones/lineages that will then be enriched by a (3) single sample clustering that (4) merges alterations that were initially negatively selected. (5) All potential phylogenies are generated and assessed for compatibility according to Definition 1. Finally the set of consolidated phylogenetic structures over time or site are output with likelihood scores
  - The pseudoclones are preserved in the samples, albeit with some dynamics (Assumption 3(2)). We carry out a multi-dimensional clustering, across all samples, based on the values or distributions of the alterations.
  - We appropriately merge the clusters of the above two steps to obtain the pseudoclones based on the similarity between the pseudoclone prevalence of the existing, multi-dimensional clusters from *step b* and with the clusters from *step a* in the appropriate samples.
2. **Phase II**. We first deduce the phylogenies of each sample separately and then we relate them with each other.
  - The sizes of the pseudoclones in each data point admits possibly multiple phylogenies. We enumerate the admissible phylogenies associating a probability with each based on Assumption 2.
  - Next we consolidate the trees from the multiple data points. This captures the topology as well as the clonal dynamics.
  Concerti offers two types of visualizations: one that captures the change-in-composition dynamics of the clones (as a tree) and the other that captures the change-in-size and birth/death of clones (fishplots [32]). The multiple possible solutions suggested by the data is output in decreasing order of probabilities to ease interpretation.

**Exception Handling**. Real data is sometimes notoriously perplexing, either due to errors in sequencing or CCF distribution estimation or simply the infraction of some of the assumptions enumerated in the last section. In practice, for intractable cases we handle the exceptions by relaxing the minimum of assumptions by consulting with the domain experts.

### Phase I: generate pseudoclones/Sublineages

We define the distance between a pair of CCF distribution *g*_1_ and *g*_2_ as

$$\text{dis}(g_{1},g_{2}) = \int_{-\infty}^{+\infty} | g_{1}(r)-g_{2}(r)| dr. $$

However, for algorithmic efficiency, we use the similarity measure defined as

$$\text{sim}(g_{1},g_{2}) = 1 - \frac{1}{2} \text{dis}(g_{1},g_{2}). $$

Thus identical distributions have a similarity of 1 while distinct distributions have similarity value zero. In practice, since the probability density function is specified as a discrete set of pairs of values and its probability, we compute the similarity as follows:

1 $$ \sum_{r=l}^{u} \min (g_{1}(r), g_{2}(r)),   $$

where [0<=*l,u*<=1.0] is the maximal interval where both *g*_1_ and *g*_2_ have non-zero values.

We first perform a negative selection where alterations not present in all samples are removed. Let *S*_*a*_ be the set of alterations present in all samples and *S*_*b*_ the removed alterations. We carry out a hierarchical clustering [33] of the alteration set *S*_*a*_ using the similarity function (1). We cluster the group of alterations in *S*_*b*_that are present in the same set of samples separately and then merge with the multi-dimensional clustering of *S*_*a*_ to produce the pseudoclones. The *prevalence* of a pseudoclone is approximated by the mean of the mean value of each constituent alteration (CCF) distribution. We merge the clusters from *S*_*a*_ and *S*_*b*_ to obtain the final set of pseudoclones if the similarity between the clusters is less than *thd* (e.g. in this manuscript we used *thd*=0.1) for all respective samples.

### Phase II: generate evolution tree(s) of pseudoclones/Sublineages

**Enumerate Admissible Trees.** Assume without loss of generality (WLOG) u=1.0. Prevalence values 0≤*a,b*≤1 can be viewed as some sub-intervals (*sticks*) of [0,1] of lengths *a* and *b* respectively. Then in a cell-population realization either sticks *A* (with prevalence value *a*) and *B* (with prevalence value *b*) are nested or disjoint but may not straddle. To remove possible computational artefacts, pseudoclones with prevalence *v*<0.05 and less than 3 SNVs for all samples are discarded. Finally, given a pseudoclone A with a prevalence value *a*, then for each *x* pseudoclone nested directly in A with prevalence *v*_*x*_, the sum of their prevalence is $\sum _{x} v_{x} \leq a $. Figure 1 shows a simple example. With bulk-sequencing (i.e. no viral isolates or single-cell), the multiple possible scenarios cannot really be teased apart. But using Assumption 2, the probability of each admissible tree can be estimated.

We present a recursive algorithm, called Stick-Stack (Algorithm 1), that enumerates all possible ways of stacking the sticks (or sub-intervals) corresponding to pseudoclones. The algorithm computes in steps 4-14 the probabilities of the trees using Assumption 2. In steps 15-19 all possible trees are generated as follows. Assuming we have already seen *j* pseudoclones and need to assign *j*+1, we iterate all possible *j* pseudoclones and determine whether the *v*_*j*+1_ can be added as child. If yes we adjust the prevalence of *v*_*i*_ and recursively call the algorithm to analyze the next pseudoclone. In the output of Stick-Stack (*A,B*) denotes nesting of *B* in *A*. Stick-Stack call is initiated as (1,*tr*=*∅*,*pr*=1.0,*v*_1_>...>*v*_*k*_>0) with the *n* prevalence values of the *k* pseudoclones and the output is a set of trees where each tree *tr* is a collection of (parent,child) pairs, with probability *pr*. It is easy to verify from the algorithm description below that the probabilities of all the admissible trees sum up to 1.


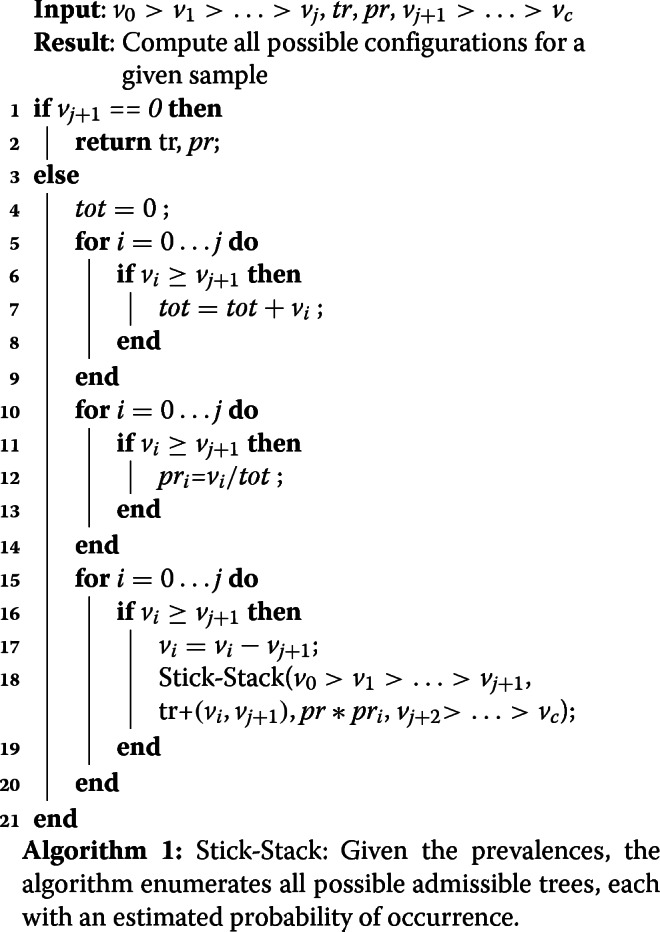


**Mutual Comparison.** While a single data point may suggest the relative relationship between pseudoclones, clonal dynamics can only be captured from multiple data points, be it multi-time or multi-site.

#### **Observation 1**

**[Clone Dynamics]** Based on Assumption 3 the clusters of filtered alterations of Step 1 of Phase I provide the clone dynamics.

- If such a cluster merges with the multidimensional cluster, then this indicates a change in composition of the pseudoclone and provides labels for the edges of the evolution phylogeny.
- If a new cluster is generated (i.e., it does not merge with clusters from the multi-dimensional clustering) then this indicates the birth of a new pseudoclone.

**Fig. 3:**
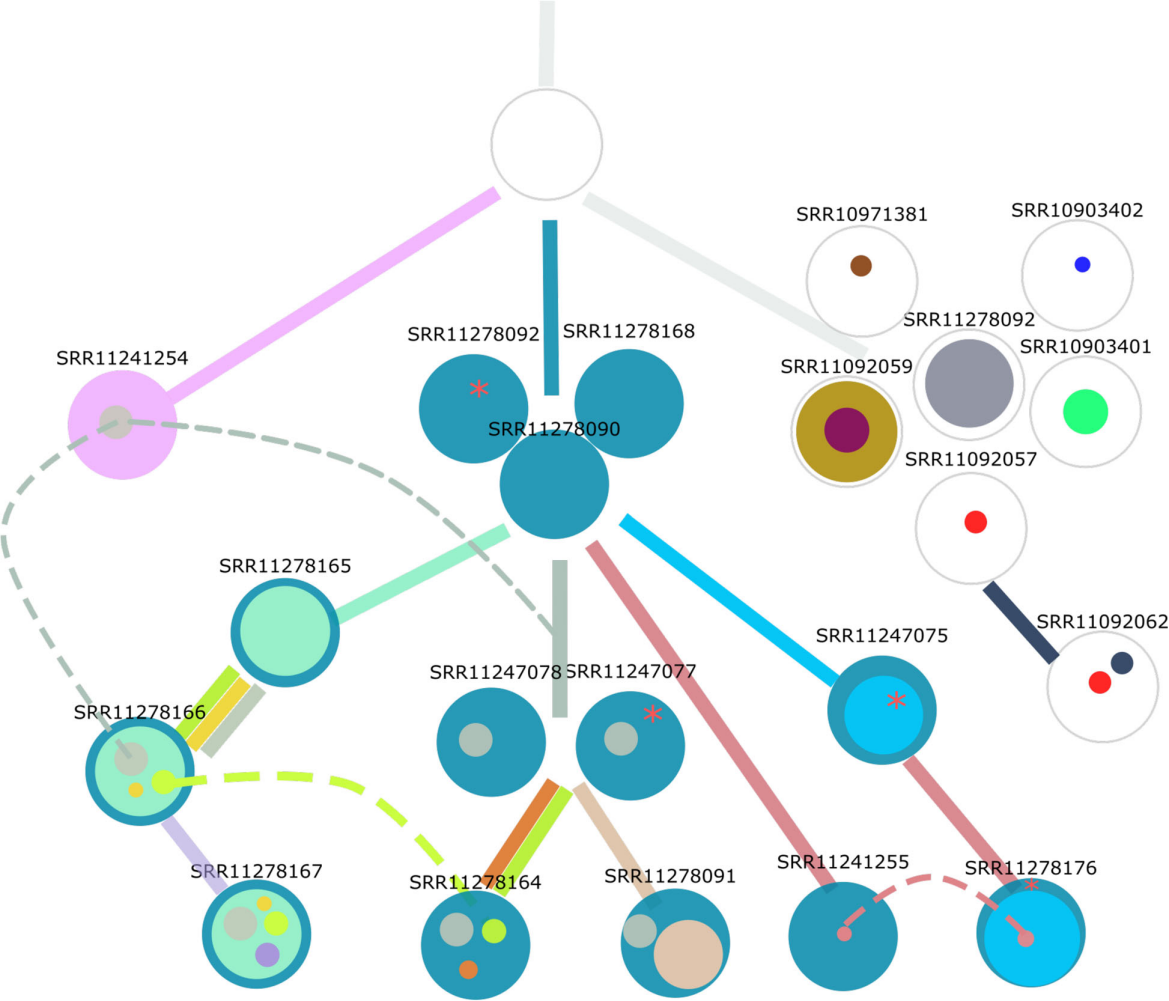
The 21 COVID-19 patients are shown at different internal and leaf nodes in the phylogeny as stacked disks of different colors. Each color corresponds to a distinct sublineage identified by a set of alterations and the size is roughly proportional to its observed prevalence value. Where possible, the edges of the phylogeny are colored by the emerging sublineage(s). When a node has multiple individuals, it indicates that there is not enough evidence to delineate the distinctions in the phylogeny. The three homoplasies (parallel mutations) are shown by dashed transversal lines. While in two (raspberry, green colors) the alteration event occurred at least twice, in the third (gray color) the alteration occurred at least three times. Furthermore, if the date of collection of a sample at a child node precedes the date at a parent node, it is within a window of a week

**Fig. 4:**
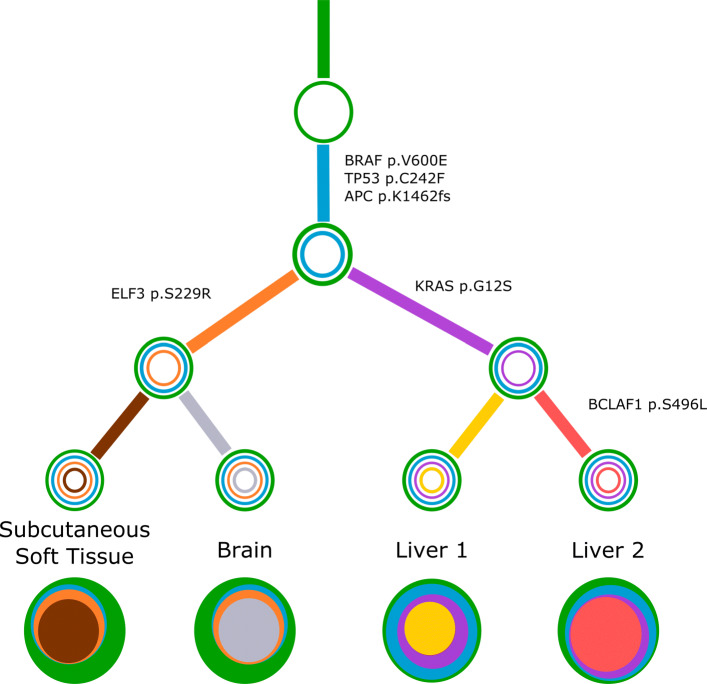
Concerti fishplot and tumor evolution tree **T** for patient CLL1 multi-time data. The fishplot width corresponds to approximate tumor size using ALC (absolute lymphocyte count) values. Clones are colored and sized proportionally to their prevalence. The corresponding tumor tree is aligned by timepoint and highlights to the birth of brown clone, which occurred prior to the annotation of clinical relapse. Node sizes correspond to prevalence. The edges of the **T** are labeled by the known cancer genes and the colors denote the distinct pseudoclones estimated by Concerti. Red asterisks indicates acquisition of new alterations to clone

**Fig. 5:**
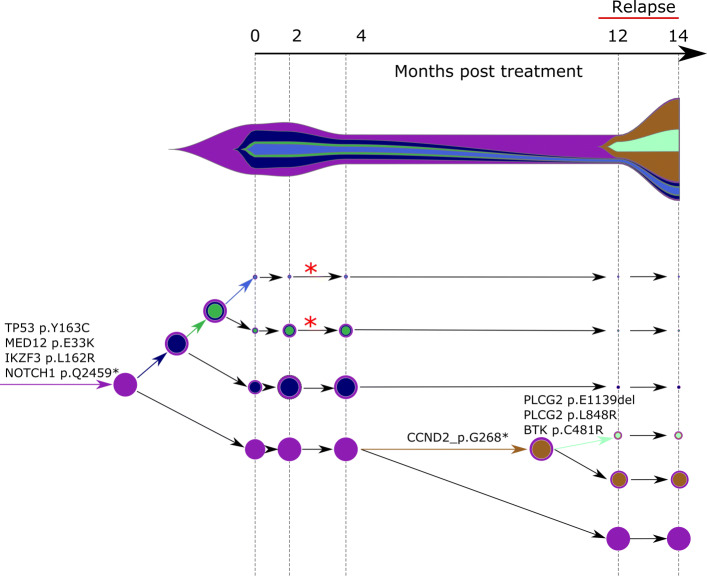
Concerti fishplot and tumor evolution tree **T** for patient CLL2 multi-time data. The fishplot width corresponds to approximate tumor size using ALC values. Clones are colored and sized proportionally to their prevalence. The corresponding tumor tree is aligned by timepoint and highlights to the birth of brown clone, which occurred prior to the annotation of clinical relapse. Node sizes correspond to prevalence. The edges of the **T** are labeled by the known cancer genes and the colors denote the distinct pseudoclones estimated by Concerti. Red asterisk indicates acquisition of new alterations to clone

A clone acquiring new alterations (case a. above) is shown as asterisk in the COVID phylogeny in Fig. 3 or tumor phylogeny in Figs. 4 and 5. The birth and death of clones (case b. above) are also illustrated in the latter two figures.

The mutual comparisons reveal whether the different samples are related or independent. When related, it is possible to reconstruct *consolidated* tree(s) that capture the evolution across the multiple data points. Stick-Stack algorithm produces each tree as a set of two-tuples corresponding to each edge as (parent,child), where the parent and child are both pseudoclones. Formally, if there exist *k*>0 parent-child pairs as (*C*_0_,*C*_1_),(*C*_1_,*C*_2_),...,(*C*_*k*−1_,*C*_*k*_), then *C*_0_*precedes* *C*_*k*_ or *C*_0_≺*C*_*k*_. Let (-,*C*_*i*_) denote that *C*_*i*_ has no parent.

#### **Definition 1**

**[Incompatible]** Let ${\mathcal {T}}_{1}$ and ${\mathcal {T}}_{2}$ be two trees with three sets of (possibly empty) pseudoclones: *A*_*i*_ that occur in both ${\mathcal {T}}_{1}$ and ${\mathcal {T}}_{2}$; *D*_*i*_ that occur in ${\mathcal {T}}_{1}$, but not in ${\mathcal {T}}_{2}$, and, *B*_*i*_ that occur in ${\mathcal {T}}_{2}$ but not in ${\mathcal {T}}_{1}$.${\mathcal {T}}_{1}$ and ${\mathcal {T}}_{2}$ are incompatible if at least one of the following conditions does not hold:

1. WLOG if *A*_1_≺*A*_2_ in ${\mathcal {T}}_{1}$ then *A*_1_≺*A*_2_ in ${\mathcal {T}}_{2}$.
2. WLOG each *D*_*i*_ is of the type (-,*D*_*i*_) and if *D*_*i*_ has a child then it occurs as (*D*_*i*_,*D*_*j*_) in ${\mathcal {T}}_{1}$.
3. WLOG each *B*_*i*_ is of the type (-,*B*_*i*_) and if *B*_*i*_ has a child then it occurs as (*B*_*i*_,*B*_*j*_) in ${\mathcal {T}}_{2}$.

Once all possible configurations of the pseudoclones for any given time point are generated independently, the next step is to extract the possible compatible trees between all time points.

#### **Definition 2**

**[Consolidated phylogeny]** Let $E({\mathcal {T}})$be the set of the two tuples (parent,child) of ${\mathcal {T}}$. If ${\mathcal {T}}_{1}, {\mathcal {T}}_{2},..., {\mathcal {T}}_{K}$ are mutually compatible, then **T**, the consolidated phylogeny of the *K* trees, is defined by the following set

$$E(\mathbf{T}) = \cup_{k=1}^{K} E({\mathcal{T}}_k). $$

Notice that by conditions 2 and 3 of the incompatible definition above, **T** does not have any nodes with multiple parents and **T** is a tree.

Let ${\mathcal {T}}_{i,j}$ be the *j*th compatible tree at data point *i* with probability *p*_*i,j*_ as estimated by Stick-Stack. Then the relative probability of a compatible evolution tree $\phantom {\dot {i}\!}\mathbf {T}^{k} = ({\mathcal {T}}_{1,j_{1}}, {\mathcal {T}}_{2,j_{1}},...,{\mathcal {T}}_{n,j_{1}})$ over the *n* datapoints is given by

2 $$ {\mathbb P}(\mathbf{T}^{k}) = \frac{p_{1,j_{1}}}{\sum_{k} p_{1,k}} \times \frac{p_{2,j_{1}}}{\sum_{k} p_{2,k}} \times \ldots \times \frac{p_{n,j_{1}}}{\sum_{k} p_{n,k}}.   $$

Note that $\sum _{k} {\mathbb P}(\mathbf {T}^k) = 1$ where *k* is over all possible compatible configurations. Thus the probability of a **T**^*k*^ may be underestimated. However, it preserves the ordering of the possible multiple solutions which is used here.

**Fig. 6:**
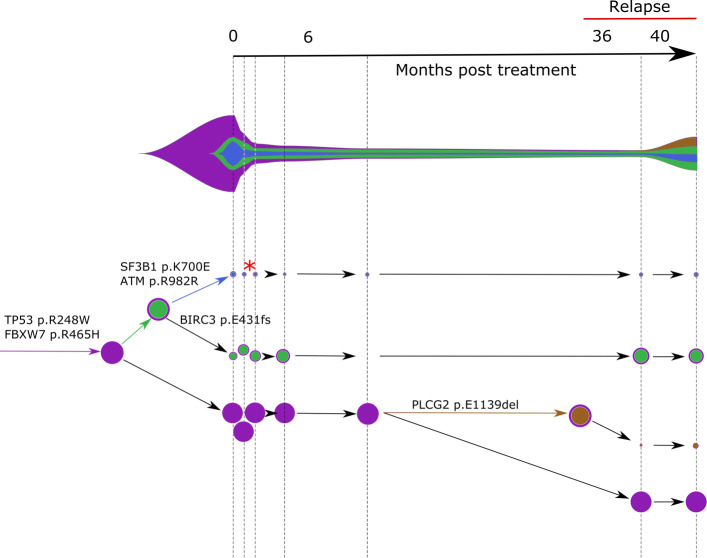
Concerti tumor evolution tree **T** for patient GI1. Tumor evolution tree **T** for colon cancer patient GI1 multi-site data. The edges of the **T** are labeled by the known cancer genes and the colors denote the distinct pseudoclones estimated by Concerti. Leaf nodes represent each of the distinct lesion sites. The single site trees $\mathcal {T}$ are shown at the bottom as stacked discs and the sizes are proportional to the prevalence values

For a concrete example, consider Fig. 6. The four sites are labeled as subcutaneous soft tissue (subcu), brain, liver1 and liver2. For each site, Concerti produces exactly one tree, with eight pseudoclones across all the four sites: green (G), cyan (C), orange (O), purple (P), ash (A), yellow (Y), red (R), brown (B). Then Stick-Stack produces the following four trees:

$$\begin{array}{@{}rcl@{}} {\mathcal{T}}_{\text{subcu}} &= &\{ \text{(G,C), (C,O), (O,B)}\},\\ {\mathcal{T}}_{\text{brain}} &= &\{ \text{(G,C), (C,O), (O,A)}\},\\ {\mathcal{T}}_{\text{liver1}} &= &\{ \text{(G,C), (C,P), (P,Y)}\},\\ {\mathcal{T}}_{\text{liver2}} &= &\{ \text{(G,C), (C,P), (P,R)}\}. \end{array} $$

It can be verified that the four trees are mutually compatible. Then the unique consolidated phylogeny is given by

$$\begin{array}{@{}rcl@{}} \mathbf{T} &= & {\mathcal{T}}_{\text{subcu}} \cup {\mathcal{T}}_{\text{brain}} \cup {\mathcal{T}}_{\text{liver1}} \cup {\mathcal{T}}_{\text{liver2}} \\ &= & \{\text{(G,C), (C,O), (O,B),(O,A),(C,P), (P,Y),(P,R)}\} \end{array} $$

**T** has only one connected component suggesting that all the data points are genetically related.

In multi-time data, a consolidated tree **T** is stretched out with the given time points appropriately marking the tree (see Figs. 4 and 5 for example). Additionally, a fishplot is output to visualize the dynamics of the pseudoclones (growth or shrinkage, including birth and death).

## Results and discussion

We applied Concerti on publicly available COVID-19 sequencing data as well as multi-site and multi-time cancer sequencing data (see Availability if data and materials section).

### COVID-19 data

For our study we sought COVID-19 patient samples with access to the raw reads in order to assess the alterations at varying allele frequencies. Using an established reference MN908947.3 obtained from the ‘first’ patient sequenced in Wuhan, China, we found 41 distinct variants in 21 patient samples (see the Availability if data and materials section for details on the patient samples and the variant calling pipeline). 18 of the patients were also analyzed in [4], albeit the variants therein were derived based on a different reference sequence and protocol. Hence, the set of variants do not exactly match the set we obtain. Our data set has three additional patients from the Wuhan seafood market [34]. We applied Concerti to the data and the resulting phylogeny is shown in Fig. 3. Note that all samples with dark blue sublineage in the figure were collected in the USA, the one with the dark-grey sublineage (SRR11278092) was collected in Nepal, while the remaining were collected in China. The figure shows the other lineages that were identified. The phylogeny is not fully resolved based on the variants of this set of patients; this is shown as clusters of patients in two internal and one leaf node in the tree. The phylogeny also uncovers three parallel mutation events (shown as dashed lines with the corresponding color): 404:A >T (raspberry), 29039:A >T (grey) and 4229:A >C (green). The first two were reported in [4] while the third is discovered in this study.

### Cancer data

Using Concerti, we analyzed three patients,two sampled over time (Figs. 4 and 5) one sampled across multiple sites (Fig. 6). We first applied Concerti to longitudinal sequencing data from two relapsed chronic lymphocytic leukemia (CLL) patients. Patient CLL1 had five biopsies taken over the course of treatment with ibrutinib and rituximab and relapsed 12 months after treatment initiation (Fig. 4). Before treatment, the dominant clone contained mutations in several known cancer genes including TP53, MED12, IK2F3, and NOTCH1. Two small clones (red asterisks) continued to evolve as evidenced by the acquisition of additional mutations after two months post-treatment. The fishplot highlights the correspondence between the emergence of a resistant clones and the increase in tumor size. Concerti’s time-scaled phylogenetic tree and fishplot captures the birth of this clone, before relapse was clinically documented, that harbored three mutations in genes associated with resistance to ibrutinib, including BTK, PLCG2, and known cancer driver CCND2.

The second CLL patient Concerti analyzed was similarly treated with and developed resistance to ibrutinib (Fig. 5). For patient CLL2, seven blood biopsies were taken over the treatment course including before-treatment, on-treatment, and at time of relapse. Several truncal mutations in known cancer genes were identified in the pre-treatment samples, including TP53 and FBXW7. After initiation with ibrutinib, a clone with a BIRC3 mutation (green) increased in prevalence. At the time of relapse, Concerti’s phylogenetic tree identifies the emergence of a new clone harboring a mutation in PLCG2, a known mechanism of resistance to ibrutinib therapy, and which goes on to grow in prevalence. Clones with ATM and SF3B1 did not have noticeable clonal dynamics during the treatment or relapse intervals suggesting they are not selected for under ibrutinib therapy. The interested reader is referred to Additional file 1 for a comparison between Concerti, CITUP [18], and Calder [25] where we show how Concerti outperforms the other methods for these two patients. In both CLL patients, the birth of these resistant clones in response to treatment was only able to be identified because of Concerti’s unique integration of time-scaled trees.

We then applied Concerti to a multi-site case, GI1, a 53 year old male with metastatic colon cancer who was part of a rapid autopsy study where multiple metastatic samples were taken at the time of death. Additional clinical details and the description of the sequencing method can be found in [7]. Samples were taken from different anatomical sites including lesions in the liver, brain, and subcutaneous soft tissue. Time of lesion development was not documented radiologically and thus no longitudinal time-ordering of the samples could be performed. Concerti’s generated tumor evolution tree gives a clinically plausible explanation as to the mutational development of this disease and is supported by the original study’s PhylogicNDT trees and their clinical findings offering a measure of validation (Fig. 6). The phylogenetic tree characterizes several truncal clones shared across all samples (green and cyan) and then identifies two sibling clones (orange and purple) that are tissue specific. One clone captures both liver samples and contains the KRAS p.G12S allele. The other clone, which contains the ELF3 p.S229R allele, goes on to develop two daughter clones each specific to the brain or subcutaneous soft tissue. Thus, Concerti’s integration of multi-site samples enables the phylogenetic tree to capture a tumor’s broad spatial heterogeneity and allows for a treatment course to be designed to be locally or broadly targeted.

## Conclusion

In this paper we introduce Concerti, an algorithm for inferring evolutionary phylogenies. Concerti’s ability to extract and integrate information from multi-point, whether multi-site, multi-time, or combination thereof samples, enables the discovery of clinically plausible phylogenetic trees that capture the heterogeneity known to exist both spatially and temporally. These models can have direct therapeutic implications since they can highlight: “births” of clones that may harbor resistance mechanisms to treatment, “death” of subclones with drug targets, and acquisition on functionally pertinent mutations in clones that may have seemed clinically irrelevant. By considering a phylogenetic analysis that steps back from the original disease context, novel relationships can be discovered before re-contextualization and interpretation in the patient context and highlights a strength of Concerti’s applicability across biological contexts. We demonstrate in this paper how Concerti can be applied to any genomic sequencing dataset with varying allele frequencies, whether it be cancer or the new SARS-CoV-2 virus causing the COVID-19 pandemic, and the results can have profound disease-specific clinical implication.

Identifying the presence of multiple viral strain infecting a single host can have significant impact on how we approach treatment, vaccine development, and mitigation strategies. The results for COVID-19 patients demonstrate Concerti’s ability to distinguish between viral strains based on difference allele frequencies and discover the presence of new homoplasies. Thus, Concerti’s results addresses the overwhelming challenges researches face when developing therapeutics and may help facilitate the key to effective vaccine development. Accurately monitoring tumor evolution over the course of a disease can lead to the identification of new drug targets and therapeutic approaches that can stabilize this complex disease and manage the selective pressures introduced by treatment exposure and tumor-environment changes. These results for patients CLL1, CLL2 and GI1 demonstrate how Concerti’s specific integration of multi-point data can facilitate better treatment plans that can both be more locally targeted and optimized for treatment responsivity.

## Supplementary Information

**Additional file 1** Experimental comparison with other tumor phylogeny reconstruction methods.

## Data Availability

All data used in the paper are available with the original publications. In particular, patient CLL1 and CLL2 correspond to B06 and A43 of [28], respectively. While the GI1 colon cancer patient used for the multi-site analysis was previously published as TPS037 in [7]. The COVID-19 patient data come from 5 NCBI BioProjects: PRJNA601736, PRJNA603194, PRJNA610428, PRJNA605983 and PRJNA608651. The reads were trimmed with Trimmomatic (v. 0.39) and then mapped with bwa on MN908947.3 GenBank sequence. This sequence was taken in December from a 57 year old woman, who sold shrimp at the Wuhuan seafood market, appears to be the earliest case with COVID-19. Variant calling was performed generating mpileup files using SAMtools and then running VarScan (min-var-freq parameter set to 0.01). Finally to remove possible sequencing artifacts, we retain SNV that: show a VarScan significance p-value <0.05 (Fisher’s Exact Test on the read counts supporting reference and variant alleles) and VAF >10%, resulting in a list of 41 SNVs in 21 patients that are used in the paper. Concerti’s binaries are available at https://github.com/ComputationalGenomics/Concerti.

## Abbreviations

SNV: Single-nucleotide variant
CLL: Chronic Lymphocytic Leukemia
VAF: Variant Allele Frequency
CCF: Cancer Cell Fraction
ALC: absolute lymphocyte count

## Footnotes

1 A preliminary version of the algorithm handling only tumor phylogeny was presented at RECOMB-CCB 2020.
