# Supplementary material for: A common methodological phylogenomics framework for intra-patient heteroplasmies to infer SARS-CoV-2 sublineages and tumor clones
Source: BMC Genomics. 2021 Nov 16;22(Suppl 5):518. doi: 10.1186/s12864-021-07660-9 (PMC8596094; doi:10.1186/s12864-021-07660-9)
Supplement: Supplementary file 1 — Additional file 1 Experimental comparison with other tumor phylogeny reconstruction methods. [file 12864_2021_7660_MOESM1_ESM.pdf]

Additional File 1

Concerti infers more plausible trees on longitudinal data than existing methods

To further evaluate Concerti's performance against alternative methods, we compared its results to Calder [25] and CITUP [18]. Here we used longitudinal data from two CLL patients because they had the most detailed clinical interpretation available and time-series data presents a greater challenge in constructing time scaled trees.

As mentioned in Table 1 of the main manuscript, Calder has a limitation on the number of individual mutations present in the data (approximatively 40).To overcome this, the authors suggest using an external clustering algorithm named Absence-Aware-Clustering [25] based on Pyclone [20], to define first the clones and then be able to build a phylogenetic tree. We ran this clustering algorithm and it yielded a substantially larger number of clones than expected. For patient CLL2, 28 clones were found with 18 being composed by a single alteration and 6 others comprised of only two alterations. Clones such as these are unlikely to be meaningful. We continued running CALDER on these two patients with the GLPK as ILP solver and it was unable to find any solution.

We also compared Concerti with CITUP (v0.1.2) which allows genome scale and longitudinal analysis. CITUP was able to successfully identify a tree for both CLL patients (Fig. S1). There are several important differences between CITUP's results and Concerti's as well as with the original publication. In CLL1, CITUP identifies a sibling clone containing the PLCG2 mechanism of resistance, just as Concerti reports. However CITUP's sibling clone is incorrectly placed as being present from the first time point; in fact there were 0 reads supporting the mutation in the first three timepoints. This error likely stems from CITUP's inability to recognize the birth of the clone. In patient CLL2, CITUP offered only the simplest tree representation, where all clones are nested, that left the PLCG2 resistance mutation in the most nested clone. This configuration does not agree with either Concerti's or the clinical analysis of the original study, Landau et al [], where the clone containing PCLG2 was not only found to compete with a sibling clone containing an ATM mutation appear but also first appeared at a later time point, again highlighting possible limitations in CITUP to capture the birth of new clones. In both patients, Concerti was found to discover trees better supported by the clinical interpretations than CITUP.

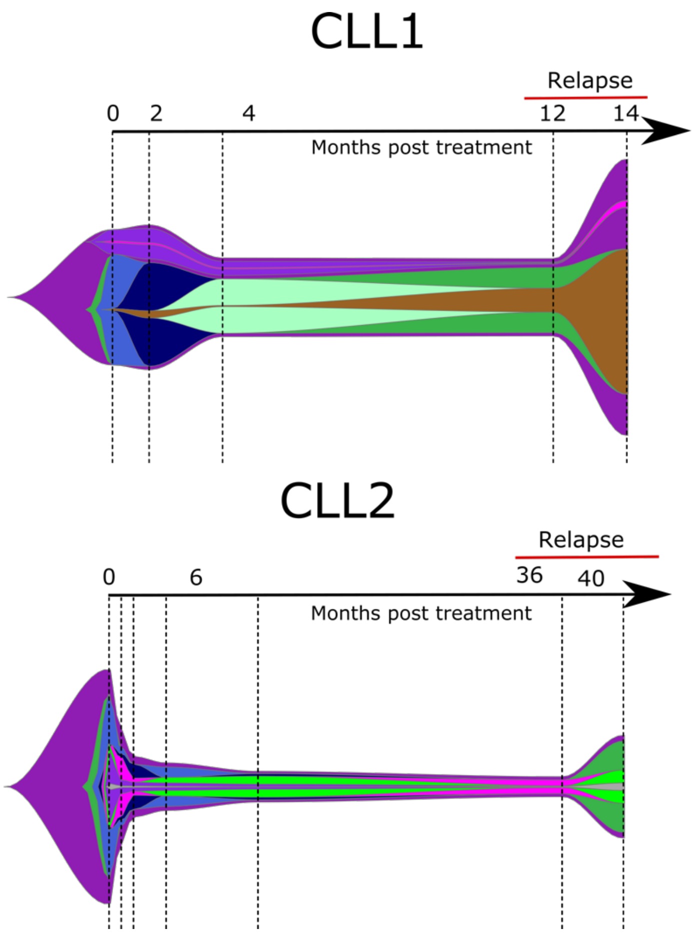

Fig S1: CLL1 and CLL2 fishplot representation identified by CITUP, width corresponds to approximate tumor size using ALC . It is worth pointing out that due to the different clonal composition it is not possible to proper match the color to the one in Figs 2 and 3.
